# Supplementary material for: CD38‐Specific Gallium‐68 Labeled Peptide Radiotracer Enables Pharmacodynamic Monitoring in Multiple Myeloma with PET
Source: Adv Sci (Weinh). 2024 Feb 29;11(16):2308617. doi: 10.1002/advs.202308617 (PMC11040352; doi:10.1002/advs.202308617)
Supplement: Supplementary file 1 — Supporting Information [file ADVS-11-2308617-s001.pdf]

ADVANCED SCIENCE
Open Access

Supporting Information

for *Adv. Sci.*, DOI 10.1002/advs.202308617

CD38-Specific Gallium-68 Labeled Peptide Radiotracer Enables Pharmacodynamic Monitoring in Multiple Myeloma with PET

*Ajay Kumar Sharma, Kuldeep Gupta, Akhilesh Mishra, Gabriela Lofland, Ian Marsh, Dhiraj Kumar, Gabriel Ghiaur, Philip Imus, Steven P. Rowe, Robert F. Hobbs, Christian B. Gocke and Sridhar Nimmagadda**

Supporting Information

for

CD38-SPECIFIC GALLIUM-68 LABELED PEPTIDE RADIOTRACER ENABLES PHARMACODYNAMIC MONITORING IN MULTIPLE MYELOMA WITH PET

Ajay Kumar Sharma[1±], *Kuldeep Gupta*[1±], *Akhilesh Mishra*[1,2], *Gabriela Lofland*[1], *Ian Marsh*[1], *Dhiraj Kumar*[1], *Gabriel Ghiaur*[3], *Philip Imus*[3], *Steven P. Rowe*[1], *Robert F. Hobbs*[1], *Christian B. Gocke*[3], and *Sridhar Nimmagadda*[1, 3, 4, 5, *]

Author affiliations:

[1]The Russell H. Morgan Department of Radiology and Radiological Science
[2]Chemical & Biomolecular Engineering, Whiting School of Engineering
[3]The Sidney Kimmel Comprehensive Cancer Center and the Bloomberg–Kimmel Institute for Cancer Immunotherapy
[4]Department of Pharmacology and Molecular Sciences
[5]Division of Clinical Pharmacology, Department of Medicine,
Johns Hopkins University School of Medicine,
Baltimore, MD, 21287, USA.
[±]Co-first authors

*Correspondence to: Sridhar Nimmagadda, Ph.D.
Johns Hopkins Medical Institutions
1550 Orleans Street, CRB II, #492
Baltimore, MD 21287
Phone: 410-502-6244
Fax: 410-614-3147
Email: snimmag1@jhmi.edu

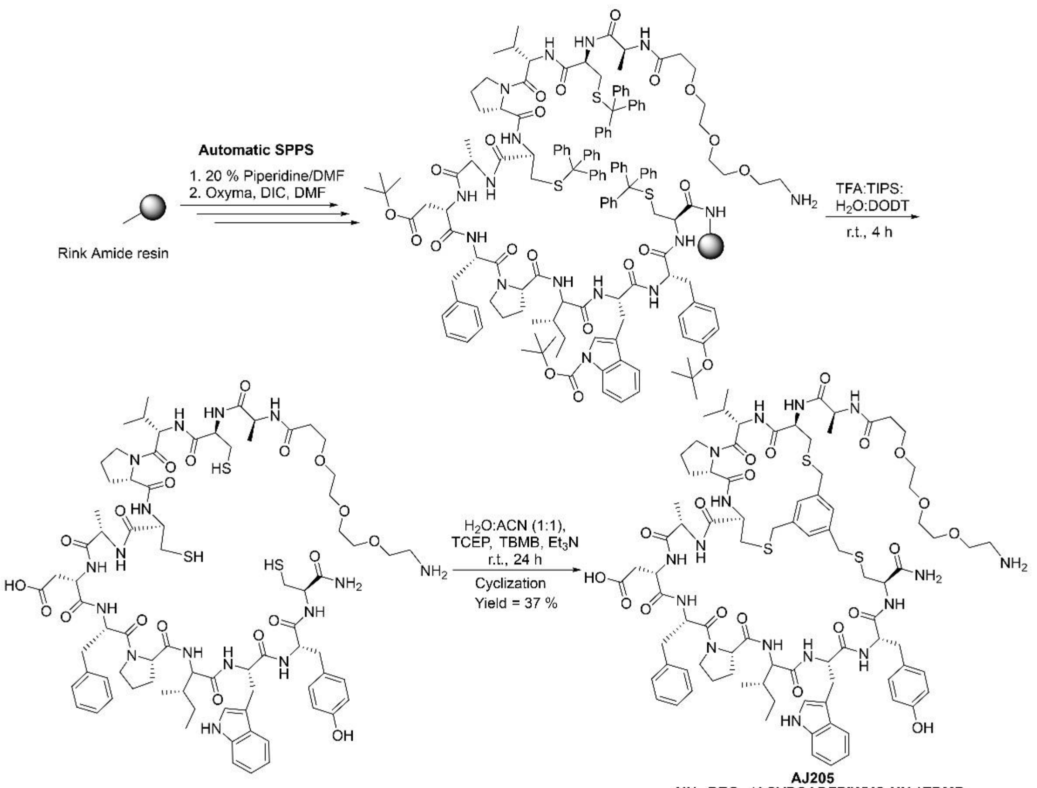

AJ205
NH_2-PEG$_3$-(ACVPCADFPIWYC-NH_2)TBMB

Scheme S1. **Synthesis of AJ205.** Automatic solid phase peptide synthesis by adding Fmoc-protected amino acids to Rink amide resin using microwave assisted coupling reaction. The created peptidyl resin was treated with cleavage cocktail to obtain deprotected linear peptide. Liner peptide was cyclized with TBMB in the presence of Et_3N in water:acetonitrile mixture to obtain AJ205.

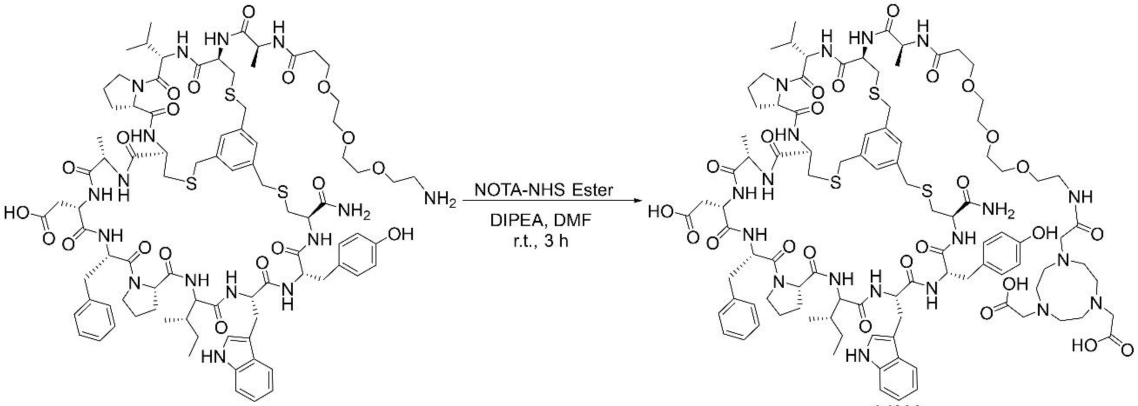

AJ205

AJ206
NOTA-PEG$_3$-(ACVPCADFPIWYC-NH_2)TBMB

Scheme S2. Synthesis of AJ206. Conjugation of AJ205 with NOTA-NHS ester in the presence of DIPEA to obtain AJ206

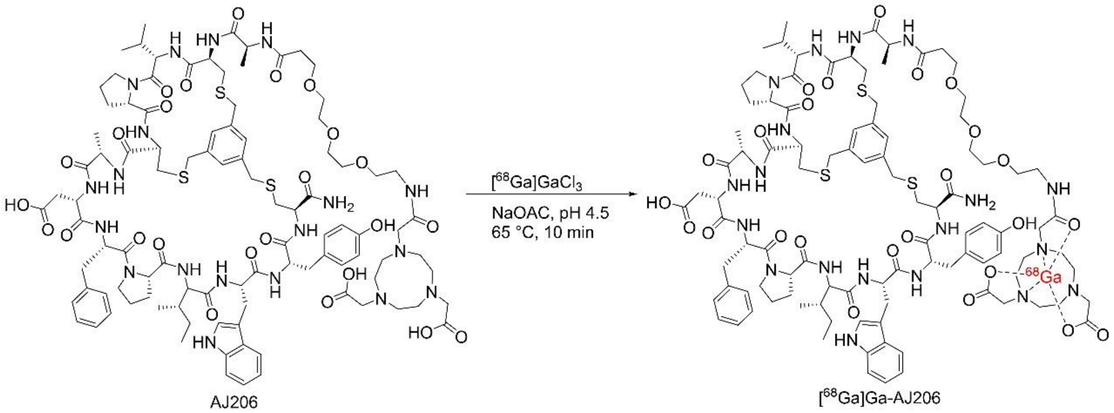

Scheme S3. Radiolabeling reaction of AJ206 with [^{68}Ga]GaCl$_3$

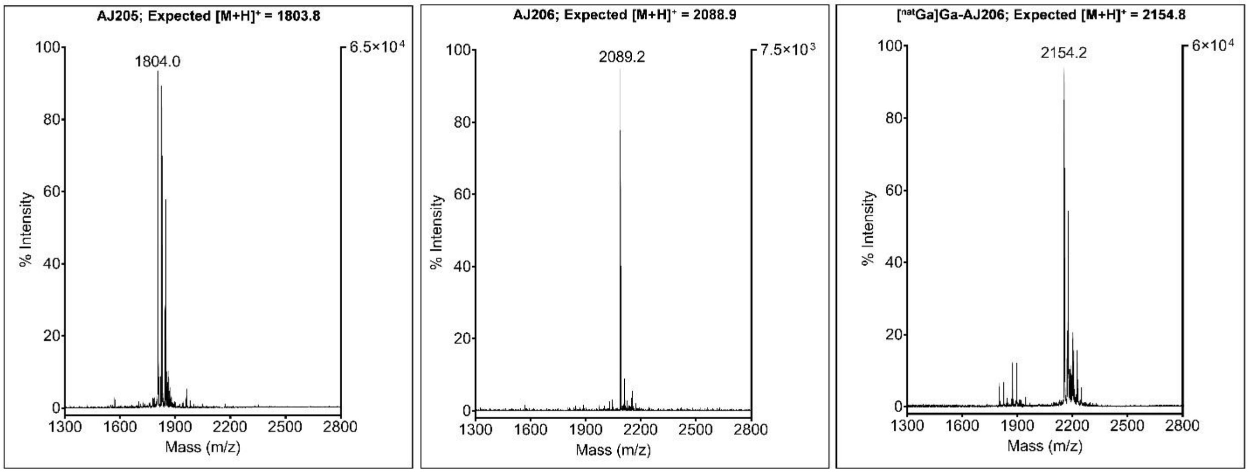

Figure S1. Characterization of AJ205, AJ206 and [^{nat}Ga]Ga-AJ206. using MALDI-TOF Mass spectrometry data confirming peptide molecular weights.

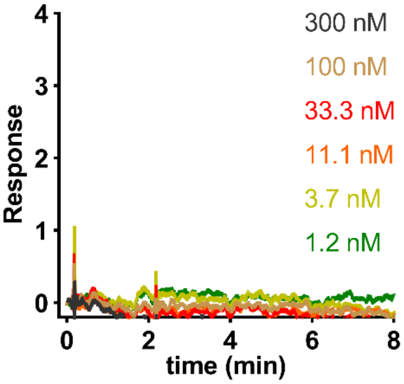

Figure S2. Surface plasmon resonance (SPR) analysis of AJ206 with purified recombinant mouse CD38 protein showing no binding to mouse CD38.

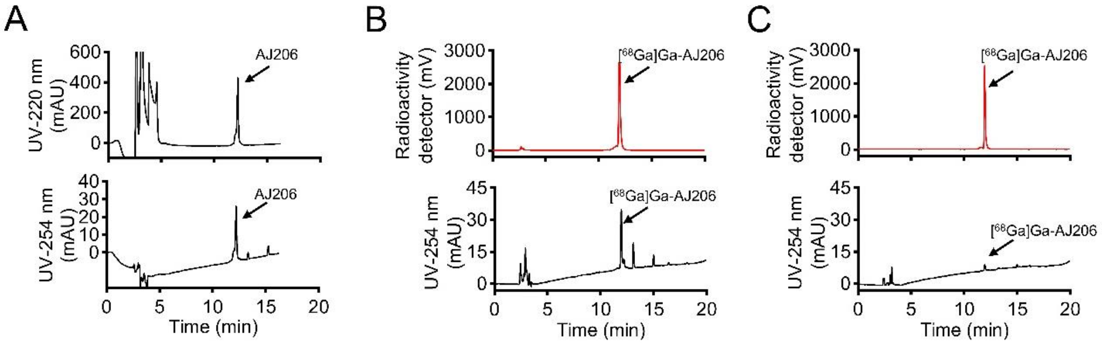

Decay corrected Radiochemical yield (RCY) = 92 ± 10.5 % (n = 35)

Radiochemical purity (RCP) = 98 ± 0.5 % (n = 35)

Specific activity = 10-15 GBq/µmol (270-400 mCi/ µmol)

Figure S3. Radiolabeling and characterization of [^{68}Ga]Ga-AJ206. **A)** HPLC chromatograms of precursor AJ206 **B)** HPLC chromatograms of reaction mixture. **C)** HPLC chromatogram of purified radiolabeled product after formulation.

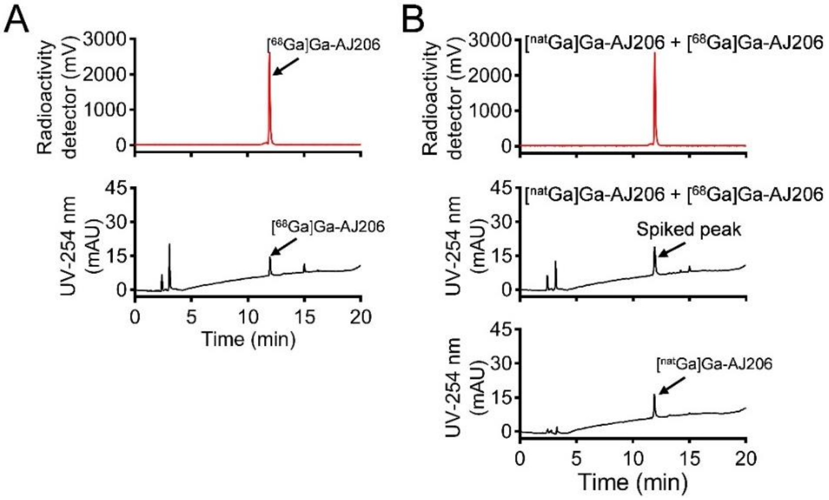

Figure S4. Characterization of [^{68}Ga]Ga-AJ206. **A)** Stability of radiolabeled product in formulation buffer at 120 min. **B)** HPLC chromatogram of chemical identity of [^{68}Ga]AJ206 with [^{nat}Ga]Ga-AJ206

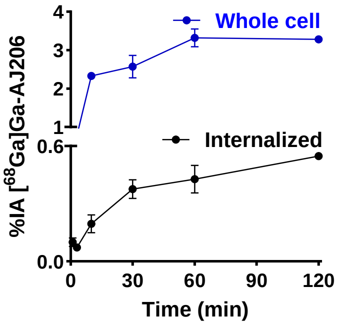

Figure S5. ***In vitro*** **binding kinetics of [^{68}Ga]Ga-AJ206 in RPMI8226 cells.** [^{68}Ga]Ga-AJ206 binding (percent incubated activity, %IA) in RPMI8226 cells to determine total cell bound and internalized activity. Cells were incubated with 5 µCi [^{68}Ga]Ga-AJ206 at 37 °C for different times until 2 hour. Cell surface receptor bound activity was washed with acidic buffer and internalized activity was quantified.

Table S1. Biodistribution of [^{68}Ga]Ga-AJ206 in mice with MM1S xenografts; data is presented as Mean±SEM (n=4) of %ID/g.

Tissues	5 min	30 min	60 min	90 min	120 min
**Blood**	20.66±1.68	2.95±0.37	0.75±0.29	0.75±0.24	0.33±0.07
**Muscle**	1.96±0.28	0.55±0.09	0.13±0.02	0.18±0.04	0.09±0.02
**Tumor**	3.09±0.16	4.15±0.53	2.48±0.17	1.60±0.46	1.30±0.40
**Thymus**	16.54±5.34	2.67±0.83	0.69±0.19	0.84±0.18	0.33±0.10
**Heart**	6.38±0.42	1.00±0.15	0.29±0.03	0.35±0.08	0.16±0.01
**Lung**	10.18±4.52	3.43±0.50	0.67±0.07	0.95±0.18	0.40±0.04
**Liver**	30.31±3.51	3.64±0.31	1.38±0.04	1.16±0.08	0.73±0.10
**Spleen**	6.41±1.30	1.21±0.15	0.41±0.05	0.43±0.07	0.28±0.05
**Pancreas**	3.55±0.34	0.79±0.14	0.19±0.03	0.26±0.06	0.17±0.05
**Adrenals**	5.07±0.11	2.08±0.38	0.59±0.06	0.44±0.10	0.57±0.14
**Kidney**	31.50±4.15	21.65±2.02	14.09±0.57	13.24±1.59	9.39±1.50
**Ovary**	4.11±1.40	1.48±0.32	0.20±0.06	0.32±0.14	0.45±0.15
**Bladder**	9.35±1.87	4.51±0.93	0.75±0.08	1.23±0.10	0.90±0.37
**Stomach (with contents)**	1.65±0.19	0.54±0.19	0.32±0.21	0.82±0.36	0.47±0.30
**Small intestine (with contents)**	5.00±0.70	0.95±0.10	0.20±0.10	0.41±0.08	0.52±0.21
**Large intestine (with contents)**	3.11±0.38	0.75±0.04	0.29±0.03	0.27±0.05	0.27±0.19
**Femur**	3.37±0.31	0.86±0.06	0.33±0.12	0.28±0.05	0.09±0.02
**Brain**	0.44±0.07	0.21±0.12	0.06±0.03	0.03±0.01	0.02±0.01

Table S2. Predicted time-integrated activity coefficients (TIACs) and absorbed dose coefficients of [^{68}Ga]Ga-AJ206 in human organs

Organs	TIAC (h)	Dose coeff. (rem/mCi)
Blood	0.09625	n/a
Muscle	0.04849	0.007548
Thymus	0.0002268	0.01961
Heart	0.001882	0.04995
Lung	0.01422	0.04884
Liver	0.04753	0.06142
Spleen	0.001063	0.026529
Pancreas	0.0005685	0.023791
Adrenals	0.0001171	0.03663
Kidney	0.02487	0.12691
Ovary	0.00007438	0.016613
Bladder	0.0007132	0.019869
Stomach (with contents)	0.0007474	0.020054
Small intestine (with contents)	0.004259	0.017501
Large intestine (with contents)	0.0006719	0.014652
Femur	0.003688	0.017242
Brain	0.0009592	0.003885

Table S3. [^{68}Ga]Ga-AJ206 uptake in MM xenografts with variable CD38 expression at 60 min post-injection; data is presented as mean±SEM (n=4 or 5) of %ID/g. *, tissue not available.

Tissues	U266	RPMI	MM1S	MM1S with Blocking	MOLP8
Blood	0.32±0.06	0.98±0.48	0.97±0.18	2.17±1.17	0.56±0.09
Muscle	0.08±0.01	0.49±0.07	0.13±0.02	0.21±0.07	0.14±0.02
Tumor	0.25±0.03	1.78±0.36	2.48±0.17	1.37±0.22	3.76±0.25
Heart	0.15±0.02	0.63±0.21	0.29±0.03	0.33±0.03	0.29±0.03
Lung	0.42±0.04	1.00±0.26	0.67±0.07	0.94±0.09	0.71±0.10
Liver	0.78±0.06	1.27±0.30	1.38±0.04	1.45±0.26	1.22±0.09
Spleen	0.24±0.01	1.09±0.15	0.41±0.05	0.90±0.45	0.39±0.03
Pancreas	*	0.48±0.15	0.19±0.03	0.64±0.39	*
Kidney	21.51±2.68	17.81±3.48	14.09±0.57	15.91±1.12	16.65±2.52
Stomach	0.39±0.30	0.45±0.07	0.32±0.21	1.01±0.56	0.25±0.06
Small intestine	0.16±0.01	0.59±0.25	0.20±0.10	0.87±0.50	0.65±0.09
Femur	0.13±0.02	*	0.33±0.12	0.25±0.09	0.28±0.07
Tumor/Muscle	3.43±0.28	3.72±0.73	21.17±3.40	8.31±2.41	27.12±2.73
Tumor/Blood	0.83±0.09	2.69±0.65	2.90±0.65	1.08±0.40	7.20±0.89

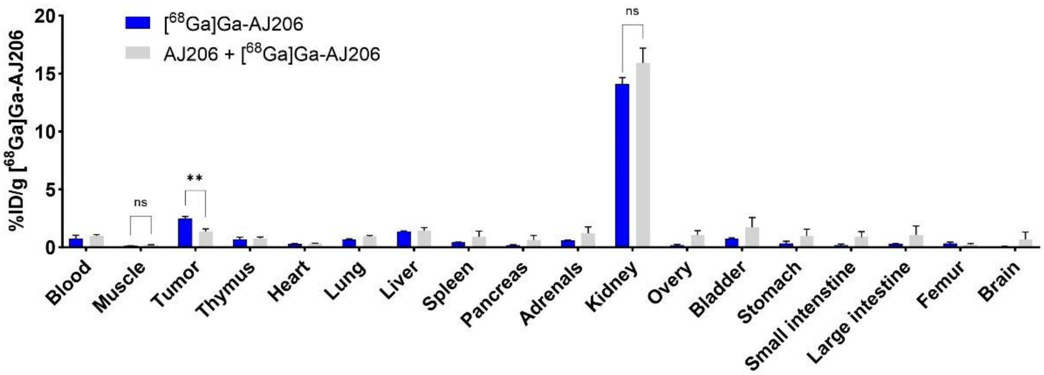

Figure S6. [^{68}Ga]Ga-AJ206 quantification by *ex vivo* biodistribution in MM1S bearing mice treated with and without a blocking dose; multiple unpaired *t* test was performed to obtain *P* values. ns, $P \geq 0.05$; ** $P \leq 0.01$.

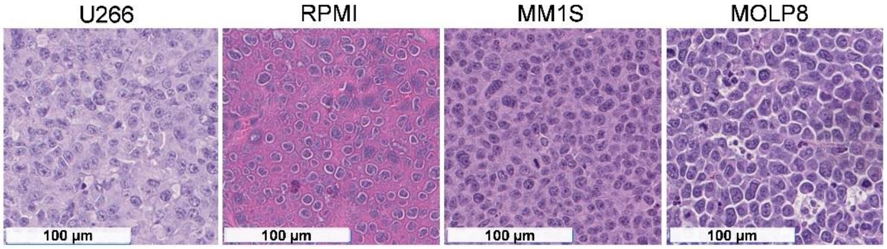

Figure S7. H&E staining of various MM tumor xenograft sections.

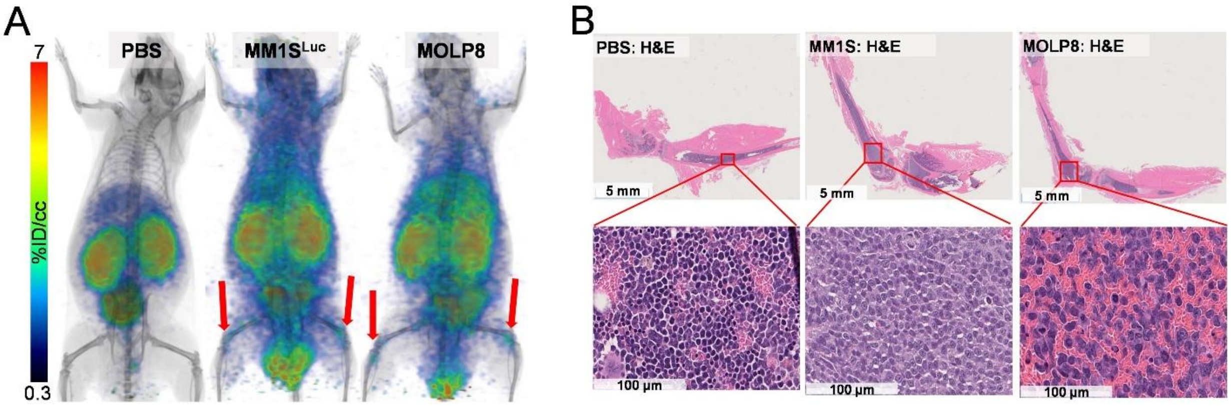

Figure S8. A) Static whole-body PET/CT images of disseminated tumor models using [^{68}Ga]Ga-AJ206. Arrow indicate tumor location in bone marrow region. **B)** H&E staining of bone section of NSG mice intravenously injected with PBS, MM1S and MOLP8 cells.

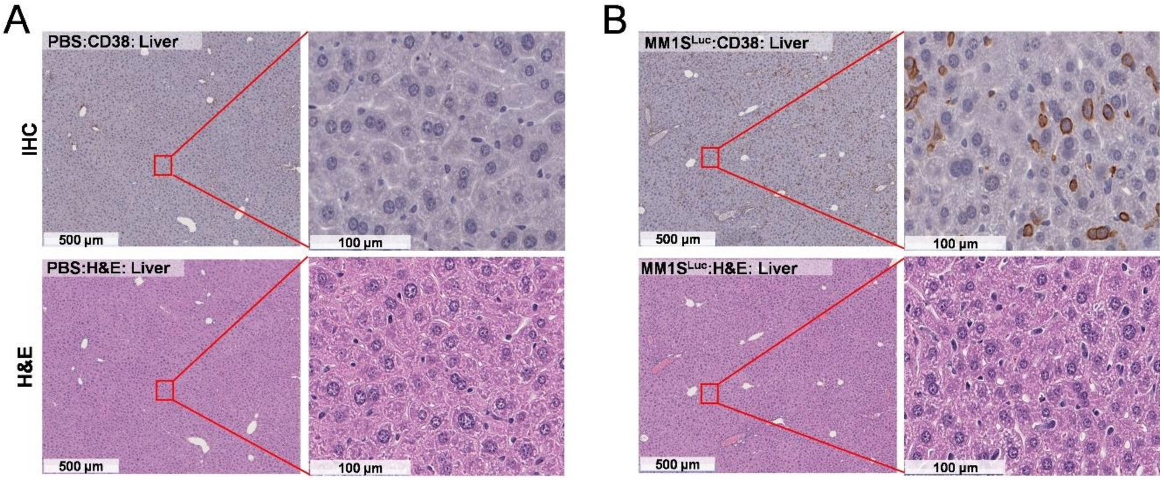

Figure S9. IHC and H&E stained slides of liver of mice intravenously injected with **A)** PBS and **B)** MM1S^{Luc} cells

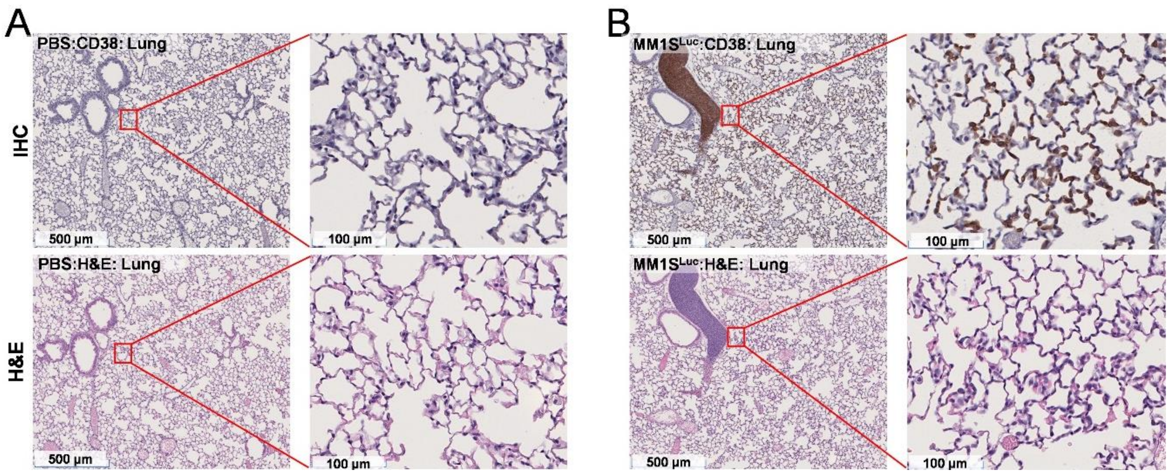

Figure S10. IHC and H&E stained slides of lungs of mice intravenously injected with **A)** PBS and **B)** MM1S^{Luc} cells

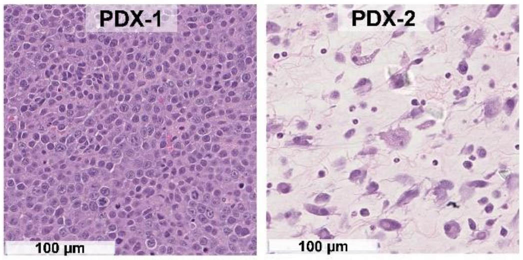

Figure S11. H&E staining of PDX-1 and PDX-2 tumor sections.

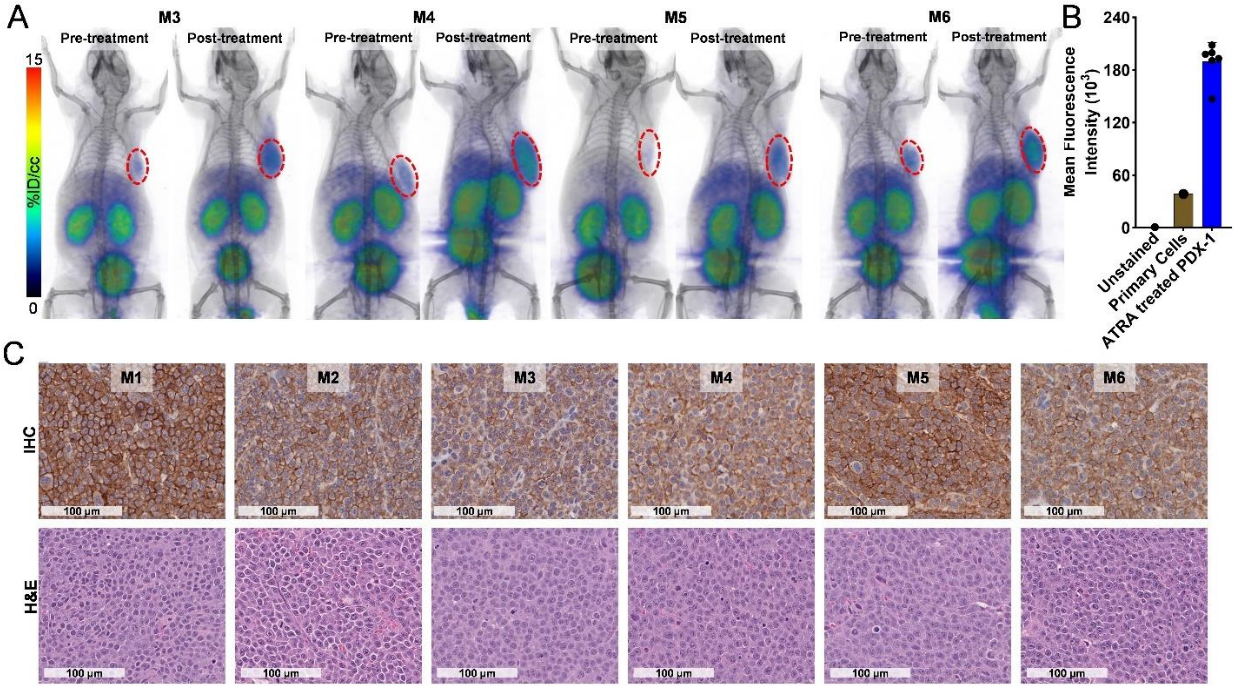

Figure S12. **CD38 pharmacodynamics imaged with [^{68}Ga]Ga-AJ206. A)** Static whole-body PET/CT scans of pre and post-ATRA treated NSG mice bearing PDX-1 at 60 min post-injection of [^{68}Ga]Ga-AJ206. **B)** Flow cytometric analysis of surface expression of CD38 receptor in primary patient cells and ATRA treated PDX-1 tumors. **C**) H&E and IHC staining slides of post-ATRA treated PDX-1 tumor sections.

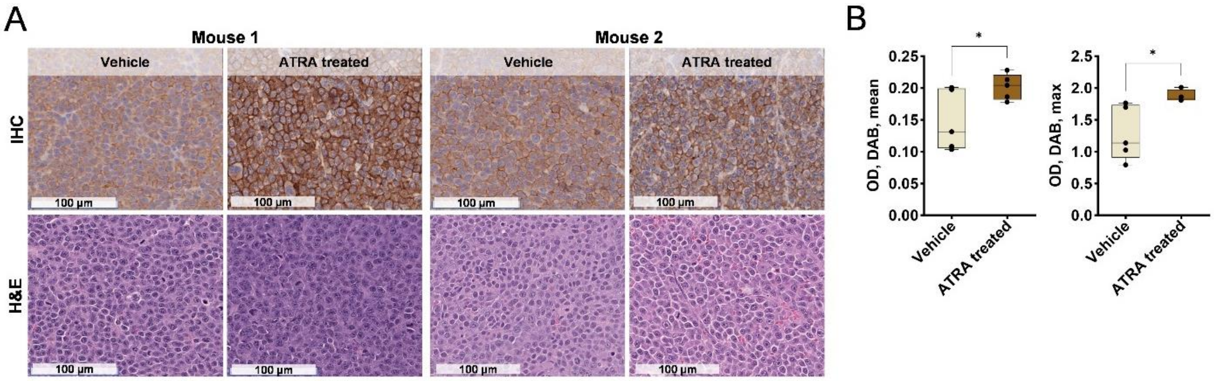

Figure S13. Histology of vehicle and ATRA treated tumor sections **A)** IHC and H&E of vehicle and ATRA treated PDX-1 tumor sections **B)** Quantification of DAB (3,3′-Diaminobenzidine) stains in IHC images showing difference between treated and ATRA treated tumor sections. This quantification was performed by Qupath software by drawing ROI on IHC of whole tumor section. Unpaired student’s *t* test was performed to obtain *P* values. *, $P \leq 0.05$.
